# Chronic hyperglycemia induces macrophage iron accumulation and promotes *Mycobacterium tuberculosis* virulence

**DOI:** 10.1016/j.isci.2026.116156

**Published:** 2026-06-05

**Authors:** Gaurav Kumar Chaubey, Rahul Dilawari, Radheshyam Modanwal, Sharmila Talukdar, Asmita Dhiman, Anil Patidar, Surbhi Chaudhary, Anurag Sindhu, Ajay Kumar, Chaaya Iyengar Raje, Manoj Raje

**Affiliations:** 1 Institute of Microbial Technology, CSIR, Sector 39A, Chandigarh 160036, India; 2 National Institute of Pharmaceutical Education & Research, Phase X, Sector 67, SAS Nagar, Punjab 160062, India; 3 Department of Biophysics Panjab University, Chandigarh 160022, India

**Keywords:** Immunology

## Abstract

Tuberculosis-diabetes comorbidity represents significant global health challenges, though the underlying mechanisms remain poorly understood. *Mycobacterium tuberculosis* (*M*.*tb*), the causative agent of tuberculosis, has a very high requirement of iron and its availability is a determining factor for successful establishment of infection. Host innate immune system and macrophages attempt to limit iron availability to restrict bacterial growth. We investigated the relationship between hyperglycemia and intracellular iron dynamics during infection using, THP-1-derived and primary macrophages from diabetic mice maintained under high-glucose conditions. Both showed increased intracellular iron along with higher expression of iron uptake receptors. Bacteria inside macrophages also contained more iron. Iron chelation significantly reduced *M*.*tb* burden in the lungs and spleen of infected diabetic mice. These findings suggest that hyperglycemia creates a “glucose legacy” that promotes iron accumulation, thereby increasing host susceptibility to *M*.*tb* infection, and reveals iron chelation as a promising adjunct therapeutic strategy.

## Highlights

• Chronic hyperglycemia induces iron accumulation in macrophages
• Chronic hyperglycemia increases iron availability for intraphagosomal bacilli
• Excess intracellular iron aids survival of infecting tuberculosis bacilli
• Iron chelation therapy ameliorates the severity of *M*. *tb* infection

## Graphical abstract

**Figure:**
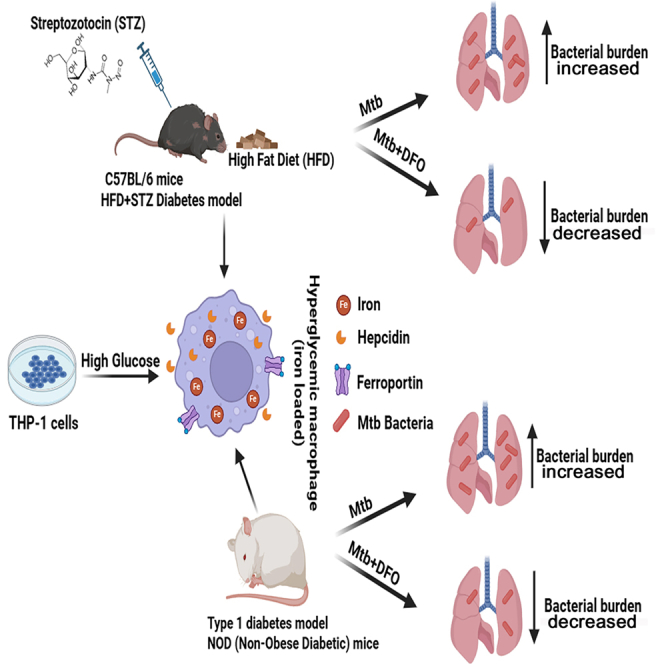

## Introduction

Diabetes mellitus (DM) is a group of metabolic disorders characterized by occurrence of elevated blood sugar levels for an extended period. Two types are commonly described, namely, type 1 and Type 2 diabetes. The latter is the most common, adult onset type of diabetes.^1^ It is a chronic inflammatory disorder in which blood glucose cannot be regulated by the body. In type 2 diabetes, the patient displays insulin resistance. Due to this sugar accumulates in the blood.^2^ Diabetes is a well-established risk factor for tuberculosis (TB).^3^^,^^4^ Its causative agent *Mycobacterium tuberculosis (M*.*tb)* is one of the most successful intracellular pathogens in terms of survival and multiplication within macrophages.^5^

Iron is an essential nutrient for all cells, and during the course of infection an intense struggle for custody of this vital resource ensues between host cells and the invading pathogenic microbes.^6^ Limiting the availability of iron is a major strategy for host defense in which most of the extracellular iron is chelated by host associated iron carrier proteins transferrin (Tf).^7^^,^^8^ During the early onset phase of infection, iron is shifted to macrophages from serum and extracellular fluids by enhanced Tf trafficking into these cells.^9^ This regulation of host cell iron storage is dependent on the nature of infection. If the pathogen resides extracellularly, then the host system attempts to create a state of hypoferremia in the extracellular milieu by locking iron within cells.^10^^,^^11^ On the other hand, in case of pathogens that reside within host cells the scenario is altered, as now these infected cells attempt to limit their own cellular iron resources from being taken over by the occupying pathogen. This is partly achieved by depleting iron from the site of infection and surrounding tissue and sequestering it into cellular storage proteins.^12^ The absorption and tissue distribution of iron is principally controlled by the interaction of the hepatic hormone hepcidin with ferroportin (FPN1). FPN1 is expressed in iron-storing and iron-transporting tissues and functions both as the hepcidin receptor and as a cellular exporter of elemental iron. The flow of iron out of the cells is controlled by hepcidin by occlusion as well as hepcidin-induced endocytosis and degradation of FPN1.^13^ In addition to systemic control of FPN1 by circulating hepcidin, FPN1 is also regulated by intracellular conditions.^14^ Earlier studies had suggested that the relocation of iron upon infection is regulated by hepcidin secreted from liver hepatocytes under the control of pro-inflammatory cytokine IL-6.^15^ More recently it has been revealed that intracellular *M*.*tb* uses the multifunctional protein GAPDH to deliver lactoferrin and Tf intracellularly to fulfill the iron requirements.^16^ Iron is crucial for *M*.*tb* virulence, serving as a cofactor for enzymes in respiration, DNA synthesis, and antioxidant defense. Host-mediated iron restriction via FPN1 and hepcidin limits bacterial growth, attenuating pathogenicity.^17^ *M*.*tb* acquires iron via siderophores (mycobactin and carboxymycobactin) that extract iron from Tf, lactoferrin, and ferritin, imported through IrtAB. It also uses heme uptake, exploits phagosomal iron, and modulates host pathways, ensuring sufficient iron for metabolism, replication, and oxidative stress resistance.^18^^,^^19^ Studies in animal models as well as patients have demonstrated that availability of an abundance of iron is favorable for the survival of *M*.*tb* inside cells.^20^^,^^21^ Upon infection, the host innate immune defense responses seek to restrict the intracellular iron stores to limit iron availability for the invading pathogen. This is achieved by a diminution of the classical iron uptake pathway coupled with enhanced cellular iron export.^22^ IFNγ regulates intracellular iron during bacterial infection by upregulating NRAMP-1 and FPN1, promoting phagosomal iron efflux and exporting iron from infected macrophages to limit pathogen survival.^23^ IFNγ also regulates the entry of iron into infected cells by down-regulating receptor-mediated endocytosis of Tf.^24^ Heparin secreted by mast cells in response to infection also directly alters intracellular iron levels in alveolar epithelial cells and macrophages by downregulating hepcidin secretion from *M*.*tb*-infected macrophages. This leads to higher FPN1 expression and an enhanced exodus of iron from infected macrophages.^25^ Diabetes is a major risk factor for TB and latent TB reactivation, with 3–4-fold increased risk, impaired glycemic control, and significantly higher mortality among TB-diabetes co-infected patients.^26^ The relationship between DM-TB has been the matter of many investigations but the underlying relationship between DM-TB is not yet completely defined.^27^ An earlier study reported a significant correlation between enhanced dietary iron intake, increased body iron stores, and the development of diabetes.^28^ A previous study revealed that high glucose altered key autophagy markers and impaired normal degradation processes, suggesting metabolic stress could compromise macrophage function and influence inflammatory responses under diabetic conditions.^29^ As iron is essential for *M*.*tb* survival, investigating this interplay with glucose regulation would assist us to understand as to how diabetes-induced hyperglycemia influences infection susceptibility and bacterial replication, thus providing a rationale for targeting iron metabolism as a therapeutic strategy in diabetic TB. We therefore established different *in vitro* and *in vivo* hyperglycemic models to test this hypothesis. In our current study, we found that chronic high-glucose exposure not only induces significant iron accumulation within macrophages but also enhances the availability of iron to intraphagosomally resident *M*.*tb* bacilli. Furthermore, systemic iron chelation markedly reduced the bacterial burden and ameliorated the severity of *M*.*tb* infection in diabetic mice, indicating that targeting iron availability can limit *M*.*tb* replication. Our findings underscore the interplay between host metabolic state, iron regulation, and bacterial virulence, providing a mechanistic basis for potential adjunctive therapies in diabetic TB patients.

## Results

### Exposure to high glucose initiates enhanced iron acquisition by macrophages

**Figure 1:**
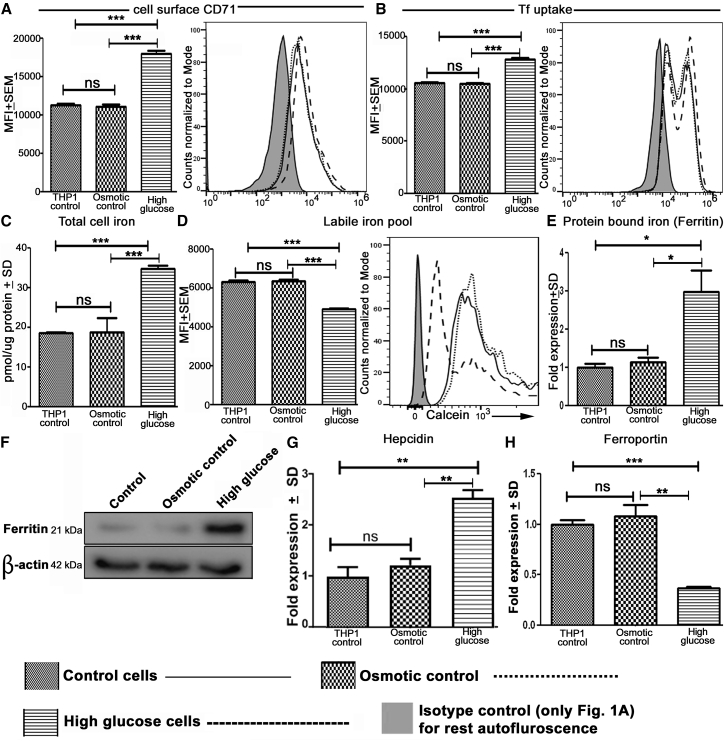
Altered surface and cytosolic expression of iron metabolism-related proteins and their effect on cellular iron in macrophages exposed to hyperglycemia *in vitro* (A) Short-term (24 h) exposure to high glucose levels *in vitro* causes a significant increase in recruitment of CD71 to the cell surface. (B) Enhanced uptake of the iron carrier Tf. (C and D) Accumulation of intracellular iron and alteration in iron import- and export-related molecules in macrophages exposed to chronic hyperglycemia *in vitro*. Elevated total iron content of hyperglycemic THP-1-derived macrophages (C), calcein quenching assay in hyperglycemic THP-1-derived macrophage indicates an elevated intracellular labile iron pool (D). The increase of cellular protein bound iron is evidenced by ferritin expression of THP-1-derived macrophage as evaluated by RT-PCR (E) and western blot of ferritin in hyperglycemic THP-1-derived macrophages (F). A significantly elevated hepcidin expression (G) coupled with a decrease in FPN1 expression (H) was observed indicating decrease in iron export from cells. All experiments were repeated independently three times. For flow cytometry experiments (A, B, and D), the representative flow cytometry histograms (right) are presented as mean fluorescence intensity (MFI±SEM) of *n* = 10^4^ cells along with data plotted as a bar graph (left, in each case). RT-PCR data presented as relative fold expression ±SD. Unpaired Student’s *t* test was used for all statistical analyses, ∗∗∗∗*p* < 0.0001, ∗∗∗*p* < 0.001, ∗∗*p* < 0.01, ∗*p* < 05, and ns = not significant. Comparison was made between cells cultured in HG medium (high glucose), with cells incubated in no glucose (NG) medium supplemented with mannitol (osmotic control), and a set of THP-1 macrophages maintained in NG medium (THP-1 control). Keys for bar graphs and flow cytometry histograms are given at the bottom of the figure.

High glucose is the hallmark of DM, resulting from impaired insulin secretion or action. Short-term hyperglycemia is clinically important because it rapidly induces oxidative stress, inflammatory signaling, and immune dysfunction. Even transient glucose spikes impair endothelial function, alter macrophage and neutrophil activity, and increase infection risk, contributing to tissue injury and predicting future metabolic and cardiovascular complications.^30^^,^^31^To evaluate the effect of acute exposure to high levels of glucose on macrophage iron metabolism, we first incubated thioglycollate (TG)-elicited macrophages for 24 h with high levels of glucose. An increased expression of the canonical Tf receptor CD71 (TFR1) on the plasma membrane of peritoneal macrophages accompanied with enhanced Tf uptake was observed Figures 1A and 1B. This suggests that acute high glucose induces an early cellular response for enhanced iron import by increase in surface recruitment of receptors for Tf internalization. Chronic hyperglycemia drives sustained oxidative stress, inflammation, and metabolic dysregulation. It causes progressive endothelial damage, immune dysfunction, leading to long-term microvascular and macrovascular complications such as neuropathy, nephropathy, cardiovascular disease, increased infection susceptibility, and organ failure.^32^ To observe the cumulative effects of long-term high glucose exposure *in vitro*, THP-1 monocytes were incubated with high glucose for an extended period over three passages (16 days). We observed a significant increase in the cells total iron content with the levels of labile iron pool and iron storage protein ferritin all being elevated (Figures 1C–1F). These cells also demonstrated significant increase in expression of hepcidin and decrease of the iron exporter FPN1 (Figures 1G and 1H). In macrophages, hepcidin binds FPN1 on the cell surface, causing its internalization and degradation. This blocks iron export, leading to intracellular iron retention. Macrophage iron storage affects immune function, inflammation, and microbial defense.

### Long-term high glucose exposure induces iron accumulation in macrophages

**Figure 2:**
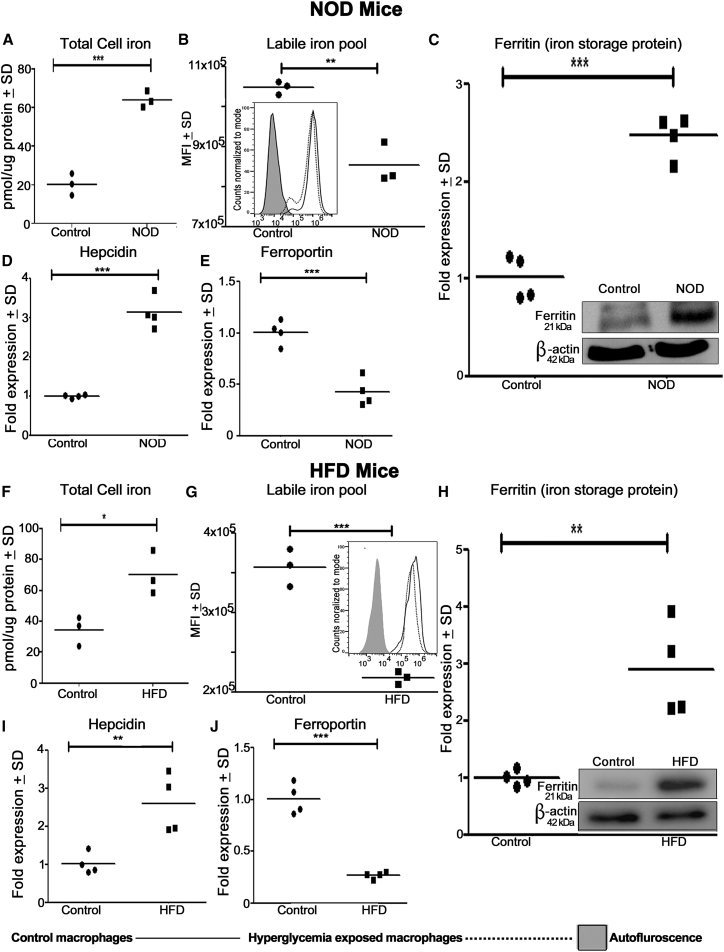
Chronic exposure to hyperglycemia results in iron accumulation within macrophages of NOD (A–E) and HFD (F–J) fed model mice Chronic high glucose induces iron accumulation in peritoneal macrophages of HFD + STZ mixed diabetic model and type 1 diabetic NOD mice. (A) Total iron content in peritoneal macrophages (PMQs) of NOD mice (*n* = 3 mice). (B) Labile iron pool status by calcein quenching assay in PMQ of NOD mice, inset represents a typical flow cytometry histogram (*n* = 3 mice). (C) Ferritin expression in PMQ's of NOD mice by RT-PCR (*n* = 4 mice), inset western blot from representative animal cells. (D) FPN1 and (E) hepcidin expression in PMQ of NOD mice (*n* = 4 mice). (F) Total iron content of long-term HFD mice peritoneal macrophages (*n* = 3 mice). (G) Calcein quenching assay of PMQ’s obtained from long-term HFD mice, the inset represents a typical flow cytometry histogram (*n* = 3 mice). (H) Ferritin expression by RT-PCR (*n* = 4 mice), inset western blot from representative animal cells. (I) Hepcidin and (J) FPN1 expression in PMQs of long-term HFD mice by RT-PCR (*n* = 4 mice). Representative flow cytometry histograms are presented as mean fluorescence intensity (MFI±SD) of 10^4^ cells, and RT-PCR data are presented as relative fold expression ±SD. Statistical analysis was performed using unpaired Student’s *t* test, ∗∗∗∗*p* < 0.0001, ∗∗∗*p* < 0.001, ∗∗*p* < 0.01, ∗*p* < 05, and ns = not significant. The key for the flow cytometry histogram is given at the bottom.

Chronic diabetes in mice models involves sustained hyperglycemia and its concomitant systemic effects. High fat diet (HFD)/Streptozocin (STZ) and non-obese diabetic (NOD) mice develop persistent inflammation, oxidative stress, immune dysfunction, and vascular injury. These physiological responses are essential for studying macrophage dysregulation, infection susceptibility, and long-term diabetic complications. Therefore, after observing the *in vitro* effects of acute and chronic exposure to high glucose on iron accumulation with macrophages in cell culture, we evaluated *in vivo* rodent models of chronic hyperglycemia. Emulating the *in vivo* results, peritoneal macrophages from both experimental animal models (long-term HFD as well as NOD mice) presented with an increased total iron content, LIP and ferritin expression. At the same time, they also demonstrated significantly elevated hepcidin expression accompanied with decrease in FPN1 (Figures 2A–2J). High iron levels in macrophages impacts infection and immune function. Excess iron fuels pathogen growth, impairing microbial clearance, and promotes oxidative stress, which disrupts cytokine production and macrophage polarization. This dysregulation weakens innate immunity, increases susceptibility to infections, and contributes to inflammation-driven tissue damage, linking iron overload to immune dysfunction.^33^^,^^34^

### The effect of exposure to high glucose on macrophage: CD71 expression, transferrin uptake, and cell iron is also observed in *M*.*tb* infected macrophages

THP-1-derived macrophages incubated in high glucose (HG) medium demonstrated significantly enhanced expression of CD71 on their surface. This was accompanied by elevated Tf internalization and an increase in total cell iron (Figures S2A–S2F).

### High glucose inhibits Nramp-1 expression in hyperglycemic macrophages contributing to elevated iron levels in intraphagosomal *M*.*tb* bacilli

**Figure 3:**
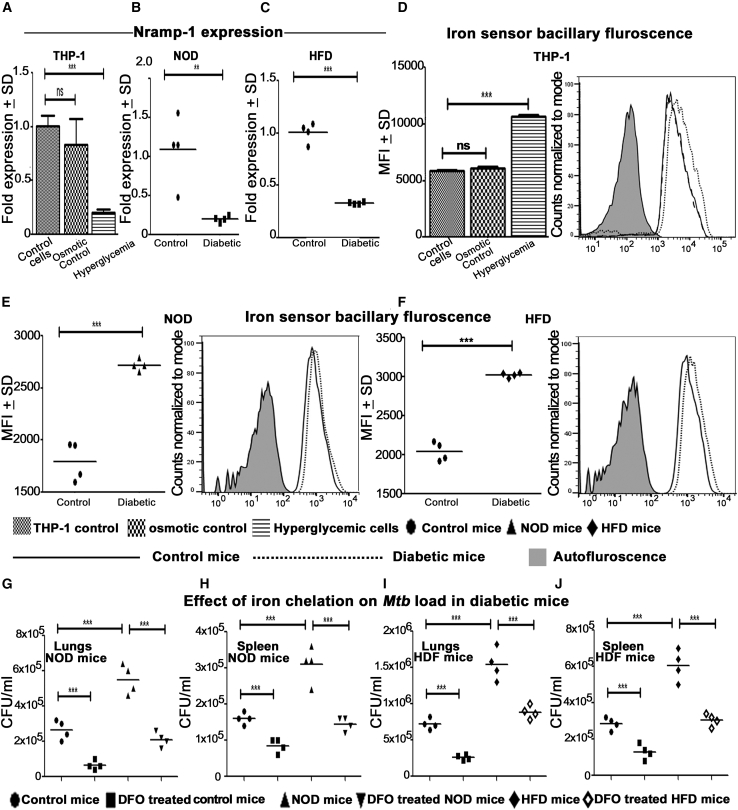
Chronic exposure to hyperglycemia leads to decreased expression of Nramp-1 in macrophages THP-1-derived macrophages (A), NOD mice peritoneal macrophages (B), and peritoneal macrophages from HFD-fed mice (C) all express significantly lower Nramp-1. RT-PCR data presented as relative fold expression ±SD. Statistical analysis was performed using unpaired Student’s *t* test, ∗∗∗∗*p* < 0.0001, ∗∗∗*p* < 0.001, ∗∗*p* < 0.01, ∗*p* < 05, and *n* = 3 independent experiments for THP-1 cells, *n* = 4 mice each for NOD and HFD mice. The availability of iron to resident intraphagosomal mycobacteria is significantly enhanced in all three cases. (D) The GFP signal (mean fluorescence intensity) of 10^4^ intraphagosomal bacilli isolated from H37Ra-bfr-GFP infected hyperglycemic THP-1-derived macrophages was recorded by flow cytometry. Data from three independent experiments is presented as a bar graph of MFI + SD (left) along with a representative flow cytometry histogram (right), ∗∗∗*p* < 0.001. (E and F) GFP signal from 10^4^ intraphagosomal bacilli isolated from H37Ra-bfr-GFP infected PMQs obtained from NOD (E) and HFD mice (F) (*n* = 4 mice in each case). At the left, data are presented as mean fluorescence intensity (MFI ± SD) for each mouse; at the right, a representative flow cytometry histogram is presented. Iron chelation significantly reduces mycobacterial load in the lungs and spleen of diabetic mice. *M.tb* colony-forming units (CFUs) from lungs and spleen of *M*.*tb* infected NOD (G and H) and long-term HFD mice (I and J) compared with CFUs obtained from matching sets of organs from DFO administered mice, *n* = 4 mice in each case. Statistical analysis was performed using unpaired Student’s *t* test, ∗∗∗∗*p* < 0.0001, ∗∗∗*p* < 0.001, ∗∗*p* < 0.01, and ns = not significant.

NRAMP-1 in macrophages exports iron and manganese from phagosomes, restricting nutrient access for intracellular pathogens like *M*.*tb*. By limiting phagosomal metal availability, it enhances antimicrobial activity playing a crucial role in host defense and controlling intracellular infections. We therefore proceeded to evaluate its expression in macrophages from all three experimental models of chronic hyperglycemia. The expression of phagosomal membrane iron exporter Nramp-1 was found to be significantly diminished in every case (Figures 3A–3C). The H37Ra bacterioferritin (bfr)-green fluroscent protein (GFP) reporter strain is an *M*.*tb* model expressing GFP under the bfr promoter, which responds to intracellular iron levels. It enables real-time monitoring of bacterial iron status within host macrophages, allowing studies of host-pathogen interactions, iron-mediated stress, and bacterial survival. This strain is valuable for evaluating therapies targeting iron metabolism and for high-throughput screening of iron-dependent bacterial responses. Utilizing this iron sensor strain to infect peritoneal macrophages we observed that the intraphagosomal bacteria had significantly elevated iron levels in all three model systems (Figures 3D–3F). This indicates that chronic hyperglycemia not only sequesters iron in the macrophage cytosol but also enhances its availability to *M*.*tb* bacilli resident inside phagosomes.

### Iron chelation decreases the *M*.*tb* load in diabetic mice

The iron chelator deferoxamine (DFO) could be a double-edged sword, potentially affecting both diabetes management and the clearance of *M*.*tb* in diabetic patients. Iron chelation has been reported to reduce *M*.*tb* bacillary burden in mice by limiting iron availability, which is essential for bacterial growth and metabolism.^35^ DFO improves insulin sensitivity by reducing cellular iron-induced oxidative stress and inflammation. Lower intracellular iron decreases reactive oxygen species, enhances insulin receptor signaling, and improves glucose uptake in tissues, mitigating insulin resistance commonly associated with obesity, high-fat diet, and metabolic disorders.^36^ We therefore proceeded to evaluate the impact of DFO on *M*.*tb* bacterial burden in diabetic mice. We infected diabetic mice with *M*.*tb* H37Rv and administered DFO treatment to these animals. Systemic treatment with iron chelator was observed to significantly reduce *M*.*tb* H37Rv load in the lungs and spleen of diabetic C57BL/6 and NOD mice (Figure 3G–3J), suggesting that DFO could serve as an adjunctive therapy for TB and could perhaps serve as an adjunct for conventional anti-TB drug regimens. DFO is known to enhance the effectiveness of bedaquiline in primary human macrophages infected with BCG.^37^ Standard anti-TB drug regimens have several limitations. Their long duration (6–9 months) often leads to poor patient adherence, increasing the risk of multidrug-resistant or extensively drug-resistant TB. Drugs like isoniazid, rifampicin, and pyrazinamide can cause hepatotoxicity, nephrotoxicity, and other adverse effects^38^ and the combination with iron chelation could boost their effectiveness and allow for a decrease in their dosage/period of administration.

## Discussion

The common exacerbating factors associated with TB include viral infection, tobacco use, old age, excessive alcohol consumption, malnutrition, and diabetes.^39^ Hyperglycemia is the most common presentation of diabetes. The increased risk of active TB among diabetics has been directly linked to severity, duration, and persistence of hyperglycemia. In the current study, we investigated the effect of diet and non-diet-induced hyperglycemia on the severity of infection in a mouse model. *M*.*tb* is an intracellular pathogen in mammals and a leading cause of death among the plethora of infectious diseases that continue to plague humankind.

To combat TB associated with morbidity, efforts across the globe are focused on targeting physiological mechanisms that can negatively influence the survival of the pathogen in mammalian hosts. The approach toward identification of novel drug targets has primarily been restricted to molecules which are exclusively targeted against the pathogen. We propose that the mechanisms that ensure iron availability to the pathogen can exacerbate the outcome of TB infection. A body of evidence suggests that the iron status of the host is a primary determinant for TB progression. Studies from sub-Saharan Africa have highlighted the relationship between iron demand and disease progression.^40^ It is well known that anemia is a common co-morbidity among TB patients. This can be due to inflammation, dietary iron deficiency, or both. Iron supplementation therapy to treat the patient’s anemia is known to lead to greater morbidity by enhancing TB disease progression,^41^ and studies have established a direct link between *M*.*tb* pathogenicity and iron accumulation.^42^ As in the case of other prokaryotes, iron has a vital role in mycobacterial physiology and its limited availability inside the host creates a nutritional stress. Upon infection with *M*.*tb*, there ensues an intense struggle between host and pathogen to withhold or acquire iron respectively. The imposition of an iron embargo serves as an important host defense strategy to arrest the survival and replication of invading pathogen.^43^ Male mice fed with high-fat diet (60% calories from fat) are useful models to mimic insulin resistance, which is one of the characterized features of type 2 diabetes.^44^ High-dose streptozotocin treatment is commonly used to induce type 1 diabetes in adult mice. It causes β-cell death due to alkylation of DNA.^45^ A low-dose STZ treatment induces a mild impairment in insulin secretion, which is more similar to the characteristics of type 2 diabetes.^46^ Based on the above line of reasoning, we utilized a diabetic model which combines HFD along with a low dose of STZ. This mimics the observed diabetes progression in many human patients.^47^

High glucose concentration in blood is the hallmark of diabetes, and increased iron levels in the body have been associated with the progression of diabetes and its resultant complications. Throughout the course of our investigation, we found that early exposure to high glucose stimulates macrophages to increase the recruitment of CD71 on their surface. This leads to increased uptake of the iron carrier Tf, which initiates the cellular iron accumulation process. Macrophages that have had a prolonged increase in iron imports will exhibit elevated iron content. This is confirmed by an increase in the metal bound to the iron storage protein ferritin and the labile iron pool. Interestingly, we observed that exposure to elevated glucose correlated with the upregulation of hepcidin and downregulation of ferroportin (FPN1), the homeostatic regulator that expels surplus iron from cells. Earlier studies have shown that an environment high in glucose raises the levels of hepcidin secreted. This is known to downregulate FPN1 at the post-translational level, which in turn prevents intracellular Fe^2+^ from being excreted out of cells.^48^ Interestingly we observed FPN1 transcription being downregulated. It could be possible that high glucose also directly regulates not only hepcidin but also FPN1 at the transcription level. Innate immunity and the regulation of iron metabolism are closely related, and hepcidin-independent regulation of FPN1 has been reported earlier. During *Salmonella* infection, macrophages have been shown to downregulate FPN1 in a hepcidin-independent manner.^49^ Another group has demonstrated that stimulation of Toll-like receptors 2 (TLR2) and 6 (TLR6) reduces FPN1 expression in mouse macrophages independent of hepcidin.^14^ The pathology associated with diabetes is mostly caused by inflammation induced by hyperglycemia, and monocytes/macrophages play a key role in coordinating these effects. Innate immunological responses and inflammation are significantly influenced by TLRs. Exposure to high glucose (15 mmol/L) has been shown to significantly induce TLR2 and TLR6 expression in THP-1 cells in a time- and dose-dependent manner.^50^ In our current study, we observed decreased expression of FPN1 gene in different high glucose models. It is conceivable that exposure to chronic hyperglycemia inhibits FPN1 gene expression via stimulation of the TLR2 and TLR6 receptors; however, further investigations regarding the exact mechanisms of this regulation are required in future.

After receiving clues from our cell culture studies, we proceeded to examine the iron status of macrophages from diabetic mice, where the cells would have been exposed to chronic hyperglycemia *in situ*. Here, we also found that macrophages accumulated iron with the metal remaining trapped intracellularly. Disruption of systemic iron homeostasis has been hypothesized to be responsible for the development of insulin resistance in diabetic patients.^51^ In addition, elevated body iron stores have been directly linked to metabolic diseases, including obesity/diabetes, dyslipidemia, and hypertension.^52^ An earlier study in endothelial cells had also reported that exposure to 25 mM glucose induces iron accumulation.^53^

In any disease state where iron accumulates in the body, the excess iron is deposited in organs throughout the body with the liver being the most notable organ with iron deposition (https://www.ncbi.nlm.nih.gov/books/NBK526131/). In both of our mouse models of diabetes, we observed iron overload in the liver (Figures S2G and S2H).

*M*.*tb* is a highly successful intracellular pathogen. To survive and replicate within host cells, it requires a continuous and copious supply of iron as a necessary micronutrient.^54^ To meet this requirement, it seeks to alter the cellular iron homeostasis to create an iron-rich intracellular microenvironment. Cellular iron content is determined not only by import of iron but also by regulation of export. It is well established that iron release from macrophages is inhibited by hepcidin, which controls iron homeostasis by influencing the degradation of the iron export protein ferroportin.^55^ An earlier study using INS-1E cell cultures revealed that glucose induces the secretion of hepcidin and insulin into the supernatant.^56^ Another group observed increased level of hepcidin in adipose tissue and serum of high-fat diet-fed mice.^57^ High glucose also induced expression of CD71 in the HK-2 cell line.^58^ Recently, Caceres and co-workers reported that a high-glucose diet in mice leads to hepatic iron overload. This iron accumulation in the liver contributes to metabolic dysfunction, linking dietary hyperglycemia with disrupted iron homeostasis and potential liver pathology in metabolic disease models.^59^ An earlier study demonstrated that iron levels modulate key mitochondrial energy pathways; increasing iron enhances citric acid cycle enzyme activities and oxidative phosphorylation, while iron depletion reduces these processes and shifts cells toward glycolysis.^60^ These reports imply that glucose may control iron levels by inducing the expression of hepcidin and iron carrier protein receptors. Nramp-1 is an antimicrobial protein that exports iron from phagosome into cytosol. It is abundantly expressed in monocytes and macrophages. These constitute the front line of the body’s immune defense against invading pathogens.^61^ Interestingly, we observed decreased expression of Nramp-1 expression in hyperglycemia-exposed macrophages, although macrophages from C57BL6/J mice have a well-described mutation which results in loss of Nramp-1 functionality.^62^ In C57BL/6 mice, the Nramp-1 gene is actively transcribed; however, a well-characterized missense mutation (G169D) results in a nonfunctional protein that is rapidly degraded. Consequently, Nramp-1 fails to properly localize to macrophage membranes, leading to impaired divalent metal (iron) transport and increased susceptibility to intracellular pathogens. In contrast, resistant strains such as 129S1 express a functional Nramp1 protein that effectively contributes to host defense. Given that Nramp-1 transcription is intact in B6 mice, we sought to investigate whether chronic high-glucose conditions influence the transcriptional capacity of the Nramp-1 gene. Hyperglycemic cells attempt to downregulate the gene; it indicates the existence of a common cellular process for keeping iron locked in. The bfr-GFP reporter strains confirmed enhanced iron availability, for *M*.*tb* residing within phagosomes, upon chronic high glucose exposure. This suggests that iron not only accumulates within host cells, but the excess iron is acquired by the resident intraphagosomal *M*.*tb*. To the best of our knowledge, the connection between high glucose and intraphagosomal iron availability has not yet been established. The increase in iron content of diabetic macrophages could be a contributing factor for elevating susceptibility to TB. The importance of iron for *M*.*tb* can be gauged from the fact that ∼40 different enzymes encoded in the *M*.*tb* genome require iron as a cofactor. It has been reported that 7 to 64 μg of Fe/g of mycobacterial cell mass is required for the proper growth of *M*.*tb* within host cells.^63^ Iron restriction inhibits the growth of many mycobacterium species.^17^ DFO is a well-studied iron chelating agent available for the effective treatment of iron overload disease like hereditary hemochromatosis.^64^ We found that treatment with the iron chelator DFO reduces *M*.*tb* load in the lungs and spleen of diabetic long-term HFD as well as NOD mice. In a recent study, we have also found that hyperglycemia affects the activation and differentiation ability of diabetic macrophages.^65^ Our current study additionally reveals that chronic high glucose exposure induces iron accumulation in diabetic macrophages, providing a rich source of essential micronutrients. Our results also suggest that iron chelation therapy could ameliorate the severity of *M*.*tb* infection in hyperglycemic individuals and provide direction for future detailed investigations in this direction.

### Limitations of the study

We would like to mention the following limitations of our current research. First, this study uses the THP-1 human macrophage cell line cultured in HG medium, HFD with STZ mice, and NOD mice as experimental model systems. These hyperglycemic models do not fully replicate the complexity of human TB-diabetes comorbidity. Human patients exhibit diverse metabolic states, genetic variability, and immune responses that may not be fully explained in these models. Second, the use of high-glucose media to mimic hyperglycemia may differ from human diabetic conditions. We know that fluctuating glucose levels are observed in diabetic patients. This limits the physiological relevance of the findings. Third, although the study shows increased intracellular iron and receptor expression, it does not fully elucidate the molecular pathways linking hyperglycemia to altered iron metabolism. Key regulators of iron homeostasis and signaling pathways remain unexplored. Fourth, this study focuses mainly on macrophages, but other immune cells, such as T cells, B cells, neutrophils, and dendritic cells, also play crucial roles in controlling *M*.*tb* infection. Fifth, iron chelation by DFO reduced bacterial burden in mice and it is used to treat iron overload disordersin human patients. However it will requireadditional clinical trials to adress issues like; possible side effects, appropriate dosing, and long-term safety in individuals with TB and diabetes before it can be applied in clinical practice.

## Resource availability

### Lead contact

Requests for further information or resources and reagents should be directed to and will be fulfilled by the lead contact, Manoj Raje (manojraje@gmail.com; rajemanoj@pu.ac.in).

### Materials availability

This study did not generate any new unique reagents. All materials used were commercially available and are listed in the key resources table.

### Data and code availability

• Data reported in this study will be shared by the lead contact upon request.
• This study does not report original code.
• All unique materials and resources used in this study, including authenticated cell lines or bacterial strains, are already listed in the key resources table and are available from the lead contact upon reasonable request.

## Acknowledgments

Mr. Anil Theophilus and Mr. Randeep Sharma are acknowledged for technical assistance. The 10.13039/501100001411Indian Council of Medical Research is acknowledged for support to M.R. by way of an Emeritus Scientist fellowship. This is IMTECH communication no. 09/2023. This research did not receive any specific grant from funding agencies in the public, commercial, or non-profit sectors. M.R. was recipient of an ICMR Emeritus Scientist fellowship.

## Author contributions

Conceptualization, M.R., C.I.R., and G.K.C.; methodology, G.K.C., R.D., R.M., and M.R.; investigation, G.K.C., S.T., A.D., A.T., S.C., A.S., A.K., and M.R.; writing – original draft, G.K.C., and M.R.; writing – review and editing, G.K.C., R.D., R.M., C.I.R., and M.R.; funding acquisition, M.R.; supervision, M.R.; project administration, M.R.

## Declaration of interests

C.I.R. has patent #Expression vector for constitutive and regulated expression of 8X-histidine tagged *M*.*tb* proteins, Indian Patent application no. 202411063076 dated 21.08.2024 pending to C.I.R., Vishant Boradia, and A.K., National Institute of Pharmaceutical Education and Research (NIPER). All other authors declare that they have no known competing financial interests or personal relationships that could have appeared to influence the work reported in this study. All the authors have no conflict of interest in reporting.

## STAR★Methods

### Key resources table

**Table:** 

REAGENT or RESOURCE	SOURCE	IDENTIFIER
**Antibodies**		

Ferritin (FTH1) antibody	Cell Signaling Technology	Cat# 3998S; RRID: AB_1903974
HRP-Rabbit Conjugated secondary antibody	Millipore Sigma	Cat# 6154; RRID: AB_258284
HRP-Mouse Conjugated secondary antibody	Millipore Sigma	Cat# A4416; RRID: AB_258167
Anti-β-Actin (ACTB) antibody	Millipore Sigma	Cat# A2228; RRID: AB_476697
APC Rat Anti-Mouse CD71	BD Pharmingen	Cat# 567258; RRID: AB_2916520
APC Rat IgG2a κ Isotype Control	BD Pharmingen	Cat# 553932; RRID: AB_479720

**Bacterial and virus strains**		

*M*.*tb* H37Rv	ATCC	TMC 102
*M*.*tb* H37Ra	BEI Resources	NR-122
*M*.*tb* H37Ra GFP	Dr Chaaya I Raje Lab (NIPER, Mohali)	N/A
*M*.*tb* H37Ra Bfr GFP	Dr Chaaya I Raje Lab (NIPER, Mohali)	N/A

**Chemicals, peptides, and recombinant proteins**		

Deferoxamine mesylate salt (DFO)	Millipore Sigma	Cat# D9533-1G
Streptozocin (STZ)	Millipore Sigma	Cat# S0130-100 mg
D-glucose	Millipore Sigma	Cat# G8270-100G
SDS	Millipore Sigma	Cat# L3771-25G
RIPA cell lysis buffer	Gbiosciences	Cat# 786-489
β-mercaptoethanol	Bio-Rad	Cat# 1610710
Protease inhibitor (EDTA-free)	Thermo Fisher Scientific	Cat# PIA32955
Blotting-Grade Blocker - 300 g nonfat dry milk	Bio-Rad	Cat# 1706404
PVDF Membrane	Bio-Rad	Cat#1620177
Holo-transferrin (human)	Millipore Sigma	Cat# T4132-100 MG
Phorbol 12-Myristate 13-Acetate (PMA)	Millipore Sigma	Cat# 19-144
Glycerol	Millipore Sigma	Cat# G5516
BD™BD BBL™ Middlebrook OADC 6x100 mL	BD Pharmingen	Cat# 212240
BD DIFCO™ Thioglycollate Medium-500g	BD Pharmingen	Cat# 243010
TRIzol™ Reagent	Thermo Fisher Scientific	Cat# 15596-026
7-AAD (7-Aminoactinomycin D)	Invitrogen	Cat# A1310
DMEM	Gibco	10566-016-500 mL
RPMI	Gibco	A10491-01 500 mL
RPMI no glucose	Gibco	11879020-500 mL
Fetal Bovine Serum	Gibco	10270106- 500 mL
Trypsin-EDTA	Gibco	25300-054- 100 mL
EDTA	Invitrogen	15575-038- 100 mL
PBS	Gibco	10010-031-1000 mL
HEPES	Gibco	15630-080-100 mL
GlutaMAX	Gibco	35050-061-100 mL
Sodium Pyruvate	Gibco	11360-070-100 mL
Hygromycin B	Invitrogen	10687010

**Critical commercial assays**		

Iron assay kit	Millipore Sigma	Cat# MAK085
RevertAid First Strand cDNA Synthesis Kit	Thermo Fisher Scientific	Cat# K1621
Pierce BCA Protein Assay Kit	Thermo Fisher Scientific	Cat# 71285-3
Molecular probe kit Alexa Fluor 647	Invitrogen	Cat# A30009

**Experimental models: Cell lines**		

THP-1	ATCC	TIB-202

**Experimental models: Organisms/strains**		

C57BL/6	Animal Facility of the CSIR-Institute of Microbial Technology (CSIR-Imtech),	N/A
Non-Obese Diabetic (NOD)	Animal Facility of the CSIR-Institute of Microbial Technology (CSIR-Imtech),	N/A

**Oligonucleotides**		

Ferritin(H), FPN1, Hepcidin, Nramp-1 and beta-actin	Millipore Sigma	See Table S1

**Software and algorithms**		

FlowJoX software	BD Biosciences	https://www.flowjo.com/solutions/flowjo/downloads
GraphPad Prism 9	GraphPad	https://www.graphpad.com/scientific-software/prism/
BioRender	Science Design Inc, Toronto, Canada	https://BioRender.com/sfw0yh5
NCBI Primer-BLAST software	NCBI, USA	https://www.ncbi.nlm.nih.gov/tools/primer-blast/

**Other**		

NanoDrop® ND-1000 Spectrophotometer	NanoDrop Technologies, DE, and USA	N/A
Accuri I C6 Flow cytometer	BD Pharmingen	N/A
FACSVerse™ Cell Analyzer	BD Pharmingen	N/A
Glucometer	AccuSure blood glucometer	N/A

### Experimental model and study participant details

#### Mice

Eight-to ten-week-old male C57BL/6 mice were procured from the experimental animal Facility of the CSIR-Institute of Microbial Technology (CSIR-Imtech), Chandigarh, India and maintained under specific pathogen-free (SPF) conditions in accordance with institutional animal care and ethical guidelines. Only male C57BL/6 mice were used in these experiments. Male C57BL/6 mice are more sensitive than females to high-fat diet-induced effects because testosterone promotes visceral fat accumulation, insulin resistance, stronger inflammatory responses and weight gain.^66^ Eight-to ten-week-old female NOD mice were also procured from the experimental animal Facility of the CSIR-Institute of Microbial Technology (CSIR-Imtech), Chandigarh, India. Diabetes incidence is higher in NOD females than in male mice. NOD mice were maintained until 20 weeks (>270 mg/dL) of age for experimental use. In this chronic model, NOD mice naturally develop autoimmune-mediated β-cell destruction, resulting in sustained hyperglycemia from 12 weeks onward, thereby closely mimicking the long-term progression of human diabetes.^67^ These models are well established and ethically approved for the study of diabetes. For iron chelation treatment, mice were injected with 100 mg/kg of Deferoxamine (DFO) for 3 days in a week for 2 weeks.^68^ All experimental protocols were approved by the Statutory Institutional Animal Ethics Committee (IAEC) vide approval No. IAEC/17/19.

#### Cell lines and primary cells

The THP-1 (TIB-202) cell line was procured from ATCC. THP-1 is a suspension cell line isolated from peripheral blood of a one-year male acute monocytic leukemia patient. Cell identity matched the supplier’s characterization, and cultures were regularly checked for normal morphology and growth behavior. Mycoplasma contamination was screened using the MycoAlert Mycoplasma Detection Kit (Lonza Cat# LT07-318) following the manufacturer’s protocol, and all tested cultures were confirmed to be negative. THP-1 Cells were cultured in RPMI-1640 supplemented with; 10% heat-inactivated FBS, 50 μmol/L β-mercaptoethanol, 2 mmol/L glutamine, 1 mmol/L sodium pyruvate, and 10 mmol/L HEPES. Before use, THP-1 cells were activated for 24 h with 25 ng/mL of phorbol 12- myristate 13-acetate (PMA) and rested for further 24h before use. Thioglycolate-elicited mice peritoneal macrophages were obtained from 6 to 8-week-old male C57BL/6 mice as described previously.^16^ Murine macrophages were maintained in DMEM media supplemented with 10% FCS.

#### Bacterial strains

*M*.*tb* H37Rv, *M*.*tb* H37Ra and *M*.*tb* H37Ra GFP were exactly as described previously.^16^^,^^69^ Th*e* H37Ra Bfr GFP strain iron sensor strain that has GFP tagged with bacterial ferritin promoter, has been described earlier; the expression of the GFP reporter is enhanced when the cells accumulate iron.^70^ All *M*.*tb* strains were cultured in Middlebrook’s 7H9 broth supplemented with 0.2% glycerol, 10% OADC and 0.1% Tween 80. The H37Ra Bfr GFP *strain* was additionally maintained under a selection pressure of hygromycin (50 μg/ml).

#### Models of high glucose exposure

Diabetes typically progresses from acute to chronic conditions through gradual metabolic and cellular changes. Initially, high glucose may be mild (acute), causing short-term disturbances in glucose homeostasis. Over time, persistent high glucose causes chronic complications such as insulin resistance, β-cell dysfunction, chronic inflammation, tissue damage, and disease susceptibility. In the current investigation we wanted to establish both acute and chronic models of exposure to elevated glucose levels to study the effects on macrophage iron metabolism. Therefore, for *in-vitro* studies on the effects of long-term exposure to high glucose, THP-1 cells were maintained in RPMI-1640 containing 5.5 mmol/L glucose (Normal Glucose control [NG]) and 33 mmol/L glucose (High Glucose [HG]) for experiments between the third and fifth passages. As an osmotic control, NG medium was supplemented with 27.5 mmol/L mannitol. To study the effect of short-term exposure to high glucose, cells were cultured in HG for 24 h along with an osmotic control. Cell viability of HG-exposed cells was confirmed to be unaffected (Figure S1A). For *ex vivo* models of macrophages isolated from diabetic mice, we utilized (i) NOD mice as a model for type 1 diabetes^71^ and (ii) For Type II diabetes model, we utilized a non-genetic rodent model of type II diabetes with a combination of insulin resistance and insulin deficiency.^47^ For these C57BL6/J male mice were fed a High Fat Diet (HFD) in which 60% of the calories were from fat for five weeks. After 5 weeks of feeding on the high-fat diet most of the animals showed signs of moderate weight gain and elevated blood glucose however, their tolerance to glucose remained comparable to that of control animals (Figures S1B–S1D). Subsequently, the animals were injected with a single low dose of streptozotocin (STZ) administered intraperitoneally (100 mg/kg). This was followed by continued HFD feeding for an additional five weeks, at which time all of them not only displayed significantly higher body weight and hyperglycemia but also glucose intolerance (fasting blood glucose ≥250 mg/dL, body weight >35 gm) (Figures S1E–S1G). These were designated as long-term HFD (LT-HFD) mice.

#### Human participants

No human subjects or primary human samples were involved in this study.

### Method details

#### Intra peritoneal glucose tolerance test (IPGTT)

The IPGTT were performed after 10 weeks of feeding mice with a high-fat diet and streptozotocin administration, as described previously.^72^ Briefly, after 6 h of fasting, mice were injected IP with 2g of glucose/kg body mass dissolved in 200 μL of sterile saline. The blood glucose levels were measured at 0, 15, 30, 60 and 120 min after I.P. injection of glucose. Blood glucose was measured using a glucometer (AccuSure blood glucometer).

#### Infection of macrophages with *M*.*tb*

PMA-activated human THP-1 cells or murine peritoneal macrophages were infected, with either, *M*.*tb* H37Rv or H37Ra strains, at a multiplicity of infection (MOI) of 1:20 and cultured as described previously.^16^ Control cells (non-infected) were cultured in parallel wherein infection with *M*.*tb* was omitted.

#### Measurement of bacterial intracellular growth

*M*.*tb* H37Rv was grown in Middlebrook 7H9 broth supplemented with 10% OADC (Difco), 0.05% Tween-80 and 0.5% glycerol to mid-log phase. For aerogenic infections, a three-jet Collision nebulizer unit (BGI, USA) was used. Briefly, 1 × 10^7^ CFU *M*.*tb* in PBS were aerosolized over a period of 15 min with approximately 90 CFU delivered to the lungs as confirmed by enumeration of bacteria on day 1 post infection. Bacterial survival and growth in tissues were evaluated after 28 days. Animals were sacrificed in CO_2_ chambers as per ethical guidelines and institutional animal care protocols. Lungs and spleen were isolated aseptically from the euthanized animals, homogenized in sterile 1× PBS and plated after serially diluting the lysate on 7H11 agar plates, supplemented with 10% OADC and antibiotics. To assess the effect of iron chelation, *M*.*tb* burden was assessed in the organs of diabetic mice treated intraperitoneally with the iron chelator deferoxamine (DFO; Sigma–Aldrich, #D9533) at a dose of 100 mg/kg, administered for 3 days per week over 2 weeks, starting 15 days post-infection.

#### Evaluation of cellular iron levels

The total iron content of 1 × 10^7^ control or hyperglycemic cells that had been harvested and washed with neutral buffer was estimated using an iron assay kit (Sigma-Aldrich, Cat: MAK085). For estimation of liver iron, samples of liver were excised from euthanized animals and rinsed with neutral buffer before iron assay. All results were presented as pmol/μg of total cellular protein; BCA method was utilized for protein estimation. The intracellular labile iron pool was assessed by flow cytometry based measurement of calcein fluorescence quenching as described previously.^73^ Macrophage stored iron pool was evaluated by western blot of cell lysates. Samples were resolved using 10% SDS-PAGE. Separated proteins were transferred onto nitrocellulose membrane and blocked using 5% casein. Blots were probed with anti-Ferritin (FTH1) antibody (#3998S CST). Primary antibody was detected with anti-mouse-peroxidase or with anti-rat-peroxidase antibodies after extensive washing with PBST and blots were developed using Luminata forte western HRP substrate (Merck Millipore).

#### Expression of Tf receptors on macrophage surface by flow cytometry

Cell membrane surface expression of receptors for the mammalian CD71 was evaluated by flow cytometry using aliquots of 5x10^5^ cells (Hyperglycemic THP-1 macrophages and peritoneal macrophages from diabetic mice) as described previously.^74^ Briefly, after washing with FACS buffer (20 mM HEPES pH 7.4, 150 mM NaCl, 1 mM CaCl_2_,1 mM MgCl_2_, 5 mM KCl, and 5% fetal calf serum), cells were blocked with FACS block (FACS buffer supplemented with 5% normal human serum and normal goat serum) at 4°C for 30 min. Subsequently, cells were incubated with anti-CD71 antibody-APC (BD #567258) and respective isotype (BD #553932) at 4°C for 1 h. After extensive washing with buffer the fluorescence signal from 10^4^ cells/sample was analyzed using FACS Verse and FACS accuri Flow Cytometers (BD). Simultaneous 7-Aminoactinomycin D (7-AAD) staining of all cell samples was carried out with data from dead cells being excluded by gating out the 7-AAD positive cells. Results were presented as mean fluorescence intensity ±standard error (MFI±SEM).

#### Labeling of transferrin

Transferrin (Sigma #T-4132) was conjugated with Alexa Fluor 647 (Molecular Probes kit #A30009) by incubating for 1 h at room temperature in 0.1 M sodium carbonate buffer (pH 9.0). Excess unbound dye was removed through extensive dialysis at 4 °C as per manufacturer’s instructions.

#### Transferrin uptake in cells

Hyperglycemic PMA-activated THP-1 cells, TG-elicited peritoneal macrophages from diabetic mice and BMDM cells were cultured in 90 mm Petri plates (5x10^6^ cells/Petri dish). After 24 h, cells were harvested with PBS-EDTA and washed with PBS. Tf uptake studies were essentially as described previously.^16^ Briefly aliquots of different cells were incubated with 5 μg Tf-A647 at 37°C for 60 min. Subsequently cells were treated with 0.1% pronase at 4°C to remove any residual surface bound Tf. Finally, cells were fixed using 4% paraformaldehyde and the samples were analyzed by flow cytometry as above.^16^

#### Isolation of intraphagosomal iron sensor bacilli

Hyperglycemic PMA-activated THP-1 cells and TG-elicited peritoneal macrophages from diabetic mice were infected with H37Ra Bfr GFP bacilli at a MOI of 1: 20 for 24 h. Subsequently, cells were harvested with PBS-EDTA, washed with PBS and processed for isolation of intra-phagosomal bacilli. Macrophages were lysed in 0.1% SDS with 1000 units/ml DNase containing SFM and centrifuged at 10,000×g for 10 min to remove lysed cellular debris. Bacteria containing pellets were washed extensively with 0.01% SDS containing SFM. Finally, samples were washed with PBS and fluorescent signal was analyzed by flow cytometry.^70^

#### Evaluation of host gene expression by quantitative RT-PCR

RNA from host cells was extracted using conventional TRIzol Reagent from treated cells.^75^ The quality and quantity of total RNA were assessed using NanoDrop ND-1000 Spectrophotometer (NanoDrop Technologies, DE, and USA). A fixed amount (100 ng) of total RNA from each sample was processed further for cDNA synthesis using RevertAid First Strand cDNA Synthesis Kit (Thermo Scientific, Massachusetts, USA) according to the manufacturer’s instructions. The RNA was converted into cDNA using Revert Aid First Strand cDNA Synthesis Kit, Thermo Scientific as per manufacturer’s instructions. RT-PCR was set as per standard conditions and protocol using gene-specific primers. The fold expression was calculated after normalizing with β-actin gene expression level for each set of treatment. The specific forward and reverse primers, designed with Primer-BLAST software, are summarized in Table S1.

### Quantification and statistical analysis

All experiments were repeated at least three to four times, and statistical analysis was performed using unpaired Student’s *t* test and one way ANOVA . Significance was defined as *p* < 0.05. Statistical annotations in figures are as follows: *p* < 0.05 (∗), *p* < 0.01 (∗∗), *p* < 0.001 (∗∗∗), and *p* < 0.0001 (∗∗∗∗). Graphs were plotted using GraphPad Prism software. FlowJo BD software was used for flow cytometry data analysis.
