# Supplementary material for: Chronic hyperglycemia induces macrophage iron accumulation and promotes Mycobacterium tuberculosis virulence
Source: iScience. 2026 Jun 5;29(6):116156. doi: 10.1016/j.isci.2026.116156 (PMC13253091; doi:10.1016/j.isci.2026.116156)
Supplement: Document S1. Figures S1, S2 and Table S1 [file mmc1.pdf]

iScience, Volume *29*

Supplemental information

Chronic hyperglycemia induces macrophage iron accumulation and promotes *Mycobacterium tuberculosis* virulence

Gaurav Kumar Chaubey, Rahul Dilawari, Radheshyam Modanwal, Sharmila Talukdar, Asmita Dhiman, Anil Patidar, Surbhi Chaudhary, Anurag Sindhu, Ajay Kumar, Chaaya Iyengar Raje, and Manoj Raje

Supplementary Material

Supplementary Figures

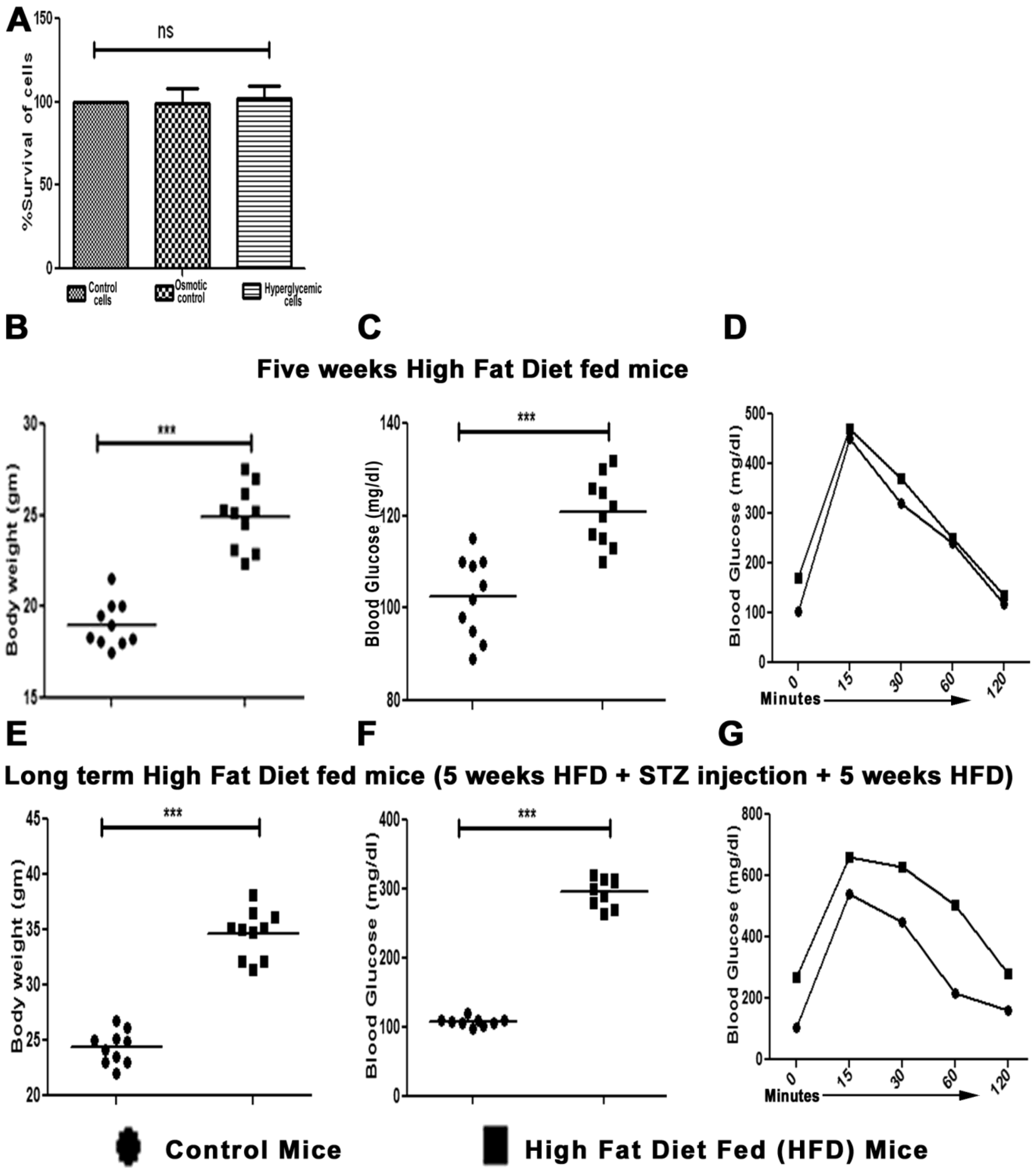

Figure S1. Effect of chronic elevated glucose exposure in cell culture and mice models. (A) Long term culture of THP1 derived macrophages in either high glucose medium or osmotic control does not affect their viability. Control cells were maintained in regular RPMI-1640 as described in methods. **(B-D)** Mice fed for 5 weeks on HFD begin to show increase in body weight and blood glucose levels though glucose tolerance remains unchanged. **(E-G)** Long Term HFD mice (5 weeks HFD + STZ + 5 weeks HFD) demonstrate significant; body weight gain, elevated blood glucose levels and glucose intolerance as compared to control mice. N=10 mice for all accept D&G where n=3, *** $p<0.001$.

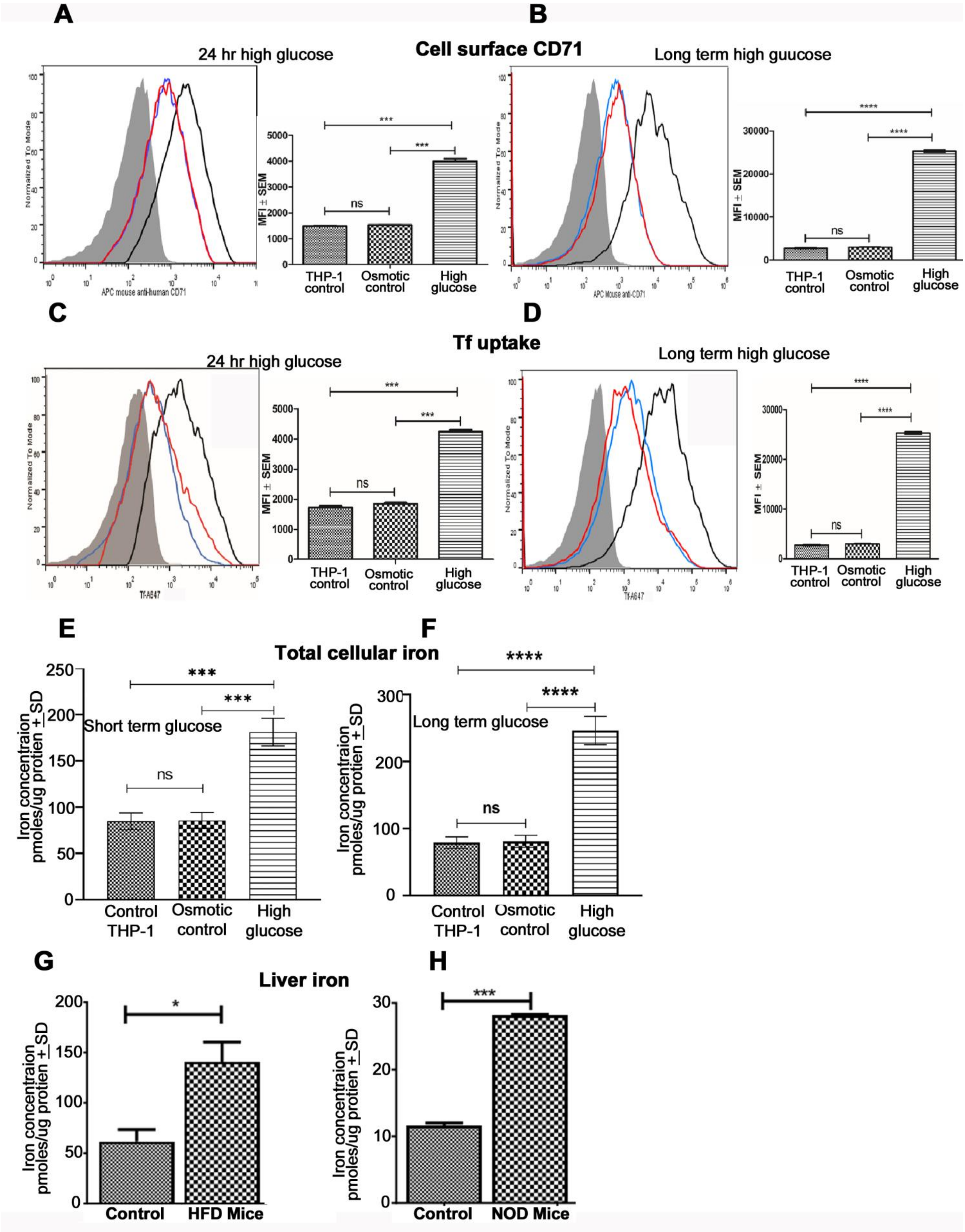
A
B
Cell surface CD71
24 hr high glucose
Long term high guucose
MFI ± SEM
THP-1 control
Osmotic control
High glucose
ns
C
D
Tf uptake
24 hr high glucose
Long term high glucose
E
F
Total cellular iron
Short term glucose
Long term glucose
Iron concentraion pmoles/ug protien +SD
Control THP-1
G
H
Liver iron
Control
HFD Mice
NOD Mice

Figure S2. The effect of exposure to high glucose on macrophage; CD71 expression, Tf uptake and cell iron is also observed in *Mycobacterium tuberculosis* infected macrophages.

The results of: elevated cell surface expression of transferrin receptor CD71 (**A&B**), Tf uptake **(C&D)** and higher total cell iron **(E&F)** is also seen in *M.tb* infected macrophages. THP-1 cells were maintained in RPMI-1640 with either: Normal Glucose control [NG], osmotic control (NG medium was supplemented with mannitol) or High Glucose [HG] for either 24hr **(A, C & E)** or 16 days **(B, D & F)**. After infection of cells with *M.tb* H37Ra-EGFP the expression of cell surface CD71 expression and cellular Tf-A647 uptake was evaluated for 10^4 infected cells/sample by flow cytometry. Representative histograms (left panel) and MFI values in bar graphs (right panel) of experiments repeated multiple times are presented. Significance was determined using Oneway ANOVA employing Tukey`s multiple comparison test (*ns p> 0.05, *** p ≤0.001*). Increase in total cellular iron in hyperglycemic macrophage infected with *M.tb* **(E&F)** and iron accumulation in liver of diabetic HFD and NOD mice **(G &H)** was evaluated by colorimetry, ***p<0.001, ** p<0.01, n=3 independent experiments **(E&F)** and n=3 mice **(G & H)**.

Supplementary Table 1

Details of primers used for analysis of gene expression

Gene	Forward primer	Reverse primer
**Human genes**		
Ferritin (H)	TGAAGCTGCAGAACCAACGAGG	GCACACTCCATTGCATTCAGCC
Ferroportin	GAGACAAGTCCTGAATCTGTGCC	TTCTTGCAGCAACTGTGTCACAG
Hepcidin	TCTGCTTTCACAGACGGGAC	CTTAGCACAGACACTCGGCA
Nramp-1	GCATCTCCCCAATTCATGGT	AACTGTCCCACTCTATCCTG
Beta-Actin	CACCATTGGCAATGAGCGGTTC	AGGTCTTTGCGGATGTCCACGT
**Mouse genes**		
Ferritin (H)	GGCTTCTGGAGATATGGTTAT	GACTCCACTGATGATTCCGATA
Ferroportin	AAGGATTGCCAGCTAACCAACA	CAGCCAATGACTGGAGAACCA
Hepcidin	GCCTGGGCACAGAGACTGAT	AAGGTGTGAGGAAAGAGCATGAC
Nramp-1	ATCCTGCCCACTGTGTTGGT	GCGAAGGGCAGCAGTAGACT
Beta-Actin	CTGCCTGACGGCCAGGT	TGGATGCCACAGGATTCCAT
